# The Interplay Between Biliary Occlusion and Liver Regeneration: Repeated Regeneration Stimuli Restore Biliary Drainage by Promoting Hepatobiliary Remodeling in a Rat Model

**DOI:** 10.3389/fsurg.2022.799669

**Published:** 2022-04-25

**Authors:** Beate Richter, Constanze Sänger, Franziska Mussbach, Hubert Scheuerlein, Utz Settmacher, Uta Dahmen

**Affiliations:** ^1^Department of General, Visceral and Vascular Surgery, University Hospital Jena, Jena, Germany; ^2^Clinic for General, Visceral and Paediatric Surgery, St. Vincenz Hospital Paderborn, University of Göttingen, Paderborn, Germany

**Keywords:** hepatobiliary remodeling, occlusive cholestasis, regeneration stimuli, biliary decompression, experimental surgery, portal vein ligation, liver resection

## Abstract

**Background and Aims:**

Patients with malignant biliary obstruction do not seem to benefit from “two-stage hepatectomy” due to an impairment of liver regeneration. We designed a novel model of “repeated regeneration stimuli” in rats mimicking a “two-stage hepatectomy” with selective or complete biliary occlusion mimicking Klatskin tumors III° or IV°. Using this new model, we wanted to investigate (1) the impact of preexistent cholestasis of different extent on the time course of liver regeneration and (2) the dynamics of hepatobiliary remodeling under regeneration conditions.

**Materials and Methods:**

Rats were subjected to a sequence of three operations: surgical induction of biliary occlusion, followed by “repeated regeneration stimuli” consisting of ligation of the left branch of the portal vein (supplying 70% of the liver volume, sPVL) as first stage and a 70%-hepatectomy (70%PHx) as second stage. Biliary occlusion (1st procedure) was induced by ligating and transection of either the common (100%, tBDT) or the left bile duct (70%, sBDT). A sham operation without ligating the bile duct was performed as control (0%, Sham). Two weeks later, on day 14 (POD14), the sPVL (2nd procedure) was performed. Another week later (POD 21), the 70%PHx (3rd procedure) took place and animals were observed for 1 week (POD 28). The first experiment (*n* = 45 rats) was dedicated to investigating liver regeneration (hypertrophy/atrophy), proliferative activity and hepatobiliary histomorphology (2D-histology: HE, BrdU) in the future liver remnant (FLR). The second experiment (*n* = 25 rats) was performed to study the dynamics of hepatobiliary remodeling in livers with different regenerative pressure (tBDT only POD21 vs. tBDT only POD 28 vs. tBDT + sPVL vs. tBDT + 70%PHx vs. tBDT + sPVL + 70%PHx) using μCT scans of explanted livers.

**Results:**

**Effect of biliary occlusion:**

Total biliary occlusion (tBDT) led to a 2.4-fold increase in whole liver volume due to severe biliary proliferation within 14 days. In contrast, partial biliary occlusion (sBDT) caused only a volume gain of the obstructed liver lobes due to biliary proliferates, resulting in a minor increase of total liver volume (1.7-fold) without an increase in bilirubin levels.

**Liver regeneration and atrophy:**

As expected, sPVL caused substantial volume gain (tBDT: 3-fold; sBDT: 2.8-fold; Sham 2.8-fold) of FLR and a substantial volume loss (tBDT: 0.9-fold; sBDT: 0.6-fold; Sham: 0.4-fold) of the portally deprived “future resected lobes” compared to the preoperative liver volume. The subsequent 70%PHx promoted a further volume gain of the FLR in all groups (tBDT: 4-fold; sBDT: 3-fold; Sham 3-fold compared to original volume) until POD 28. Hepatobiliary remodeling: After tBDT, we identified histologically three phases of hepatobiliary remodeling in the FLR. Following tBDT, biliary proliferates developed, replacing about 15% of the hepatocellular tissue. After sPVL we found incomplete restoration of the hepatocellular tissue with a visible reduction of the biliary proliferates. The 70%PHx led to an almost complete recovery of the hepatocellular tissue in the FLR with a nearly normal liver architecture. In contrast, after sBDT and Sham we observed a near normal liver morphology in the FLR at all time points. CT-scanning of the explanted livers and subsequent 3D reconstruction visualized the development of extrahepatic biliary collaterals. Collaterals were detected in 0/5 cases 1 week after sPVL (first regeneration stimulus), and in even more cases (3/5) 1 week after the 70%PHx (second regeneration stimulus). Histological workup identified the typical biliary cuboid epithelium as inner lining of the collaterals and peribiliary glands.

**Conclusion:**

Liver volume of the FLR increased in cholestatic rats mainly due to biliary proliferates. Application of repeated regeneration stimuli in the style of a “two-stage hepatectomy” promoted almost full restoration of hepatocellular tissue and architecture in the FLR by reestablishing biliary drainage via formation of biliary collaterals. Further exploration of the dynamics in hepatobiliary modeling using this model might help to better understand the underlying mechanism.

## Introduction

The impressive effect of “two-stage hepatectomy” on volume gain of the future liver remnant (FLR) in case of initially non-resectable liver tumors is well-known by now (1–5). For the small population of cholestatic patients, several authors described a minor volume gain of the FLR with a dramatically high mortality (10–18%) due to liver failure (3, 4, 6, 7). Therefore, the authors recommended that patients with systemic cholestasis should be carefully selected based on the “liver failure criteria” (e.g., systemic total bilirubin, future liver remnant ratio >0.4, preoperative prothrombin time >1.2) (3, 4, 6, 7). However, the “safe” extent of liver resection in patients with occlusive cholestasis is controversially discussed (1–4, 6, 7).

Preventing postoperative liver failure in cholestatic patients remains rather difficult. Cholestasis can lead to substantial alterations of the liver architecture. The severity is related to the duration and extent of biliary occlusion, which in turn may impair liver regeneration (8–23). Exploring the dynamics of histopathological alterations following biliary occlusion in regenerating livers might be helpful to better understand the impaired liver regeneration in occlusive cholestasis.

Therefore, we established a novel surgical model consisting of a sequence of three different hepatobiliary procedures: (1st) Ligation and transection of either the common or the left bile duct or sham operation, followed by (2nd) a 70% portal vein ligation on POD 14, and (3rd) a 70% partial hepatectomy on POD 21. With this sequence we wanted to represent the clinical situation of either a Klatskin IV° by ligation and transection the common bile duct or a Klatskin III° by ligation and transecting the left bile duct.

Using this novel model we wanted to investigate:

(1) The impact of pre-existent cholestasis of different extent on the time course of liver regeneration and
(2) The dynamics of hepatobiliary remodeling under regeneration conditions.

## Materials and Methods

### Experimental Design

We designed two experiments in male Lewis rats (*n* = 70).

***Experiment No. 1: This experiment was designed to study the impact of “repeated*** ***regeneration stimuli” after inducing biliary occlusion of different extents (n*** **=** ***45) on*** ***the course of liver regeneration and hepatobiliary remodeling***

The experiment aimed for investigation of the histopathological alterations in the portally deprived “future resected lobes” and the portally supplied future liver remnant (FLR) during the course of “two-stage hepatectomy” in different extents of biliary occlusion. Rats were subjected to a sequence of three operations: surgical induction of biliary occlusion (1st procedure), followed by a “two-stage hepatectomy” consisting of ligation of the left branch of the portal vein (supplying 70% of the liver volume, sPVL, 2nd procedure) as first stage and a 70%-hepatectomy (70%PHx, 3rd procedure) as second stage.

**Figure 1:**
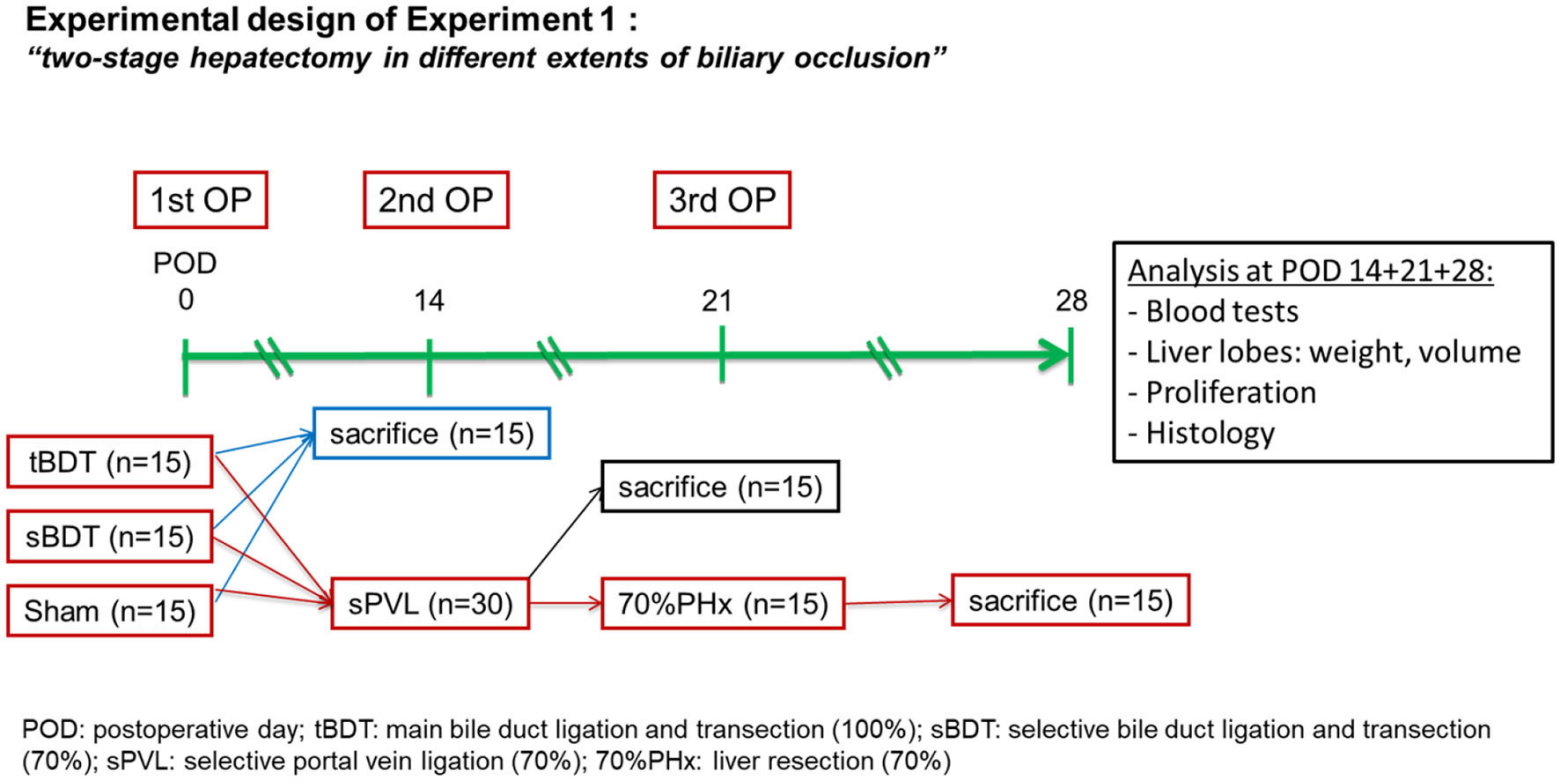
Design of Experiment 1 with the sequence of the “two-stage hepatectomy” in different extents of biliary occlusion (tBDT 100%, sBDT 70%, Sham 0%) in rats (*n* = 45). We included a group with minor (70%) and a group with total biliary occlusion mimicking Klatskin III° and IV°, respectively. Sham operated animals were used as control group. The 2nd procedure was a selective portal vein ligation (70% of liver) at POD 14, followed by a 70% liver resection at POD 21 and sacrifice at POD 28. We included three time points (POD 14, 21, 28) for investigation of systemic cholestasis (using peripheral blood), proliferative activity of hepatocytes and cholangiocytes and the histopathological alterations of the liver tissue in the “future resected lobes” (median lobe and left lateral lobe, ML+LLL) and future liver remnant (right lobes and caudate lobes, RL + CL) during the “two-stage hepatectomy”.

The first operation was the induction of biliary occlusion of different extent: 100% of liver in tBDT, 70% of liver in sBDT and 0% in Sham Figure 1.

The second procedure was the selective ligation of the left branch of the portal vein [sPVL, 70% of liver: median lobe and left lateral lobe (ML+LLL)] at postoperative day (POD) 14.

The third procedure was an extended liver resection (70%PHx, ML+LLL) at POD 21. The animals were observed until POD 28; and were sacrificed at POD 14, 21 and 28. Detailed description of the surgical procedures are given in Supplementary Material.

At POD 14 we examined the histopathological alterations after induction of different extents of biliary occlusion (tBDT with 100% or sBDT with 70% or Sham with 0%) and prior to the selective portal vein ligation (sPVL, 70% of liver volume).

At POD 21 we examined the histopathological alterations 1 week after sPVL in different extents of biliary occlusion (tBDT or sBDT or Sham) and prior to the extended liver resection (70%PHx).

At POD 28 we examined the histopathological alterations 1 week after completing the “two-stage hepatectomy” in different extents of biliary occlusion (tBDT or sBDT or Sham + sPVL +70%PHx, respectively).

***Experiment 2a-c: The experiment was designed to visualize formation of biliary collaterals by*** ***reconstructing* μ*CT-images*** ***of explanted livers after each of the three surgical procedures*.**

To detect biliary collaterals, we injected radiopaque Microfil into the dilated bile duct after sacrifice at the dedicated time points (see Supplementary Figure 1).

A biliary collateral describes a non-preexisting connection between a dilated and a non-dilated segment of the extrahepatic bile duct. The separation into two segments of the extrahepatic bile ducts is a result of the triple ligation of the extrahepatic bile duct with transection of the ligated bile duct between the two most distal ligatures (tBDT). The proximal part (near to liver) is dilated due to congested bile fluid inside the bile duct. In contrast, the distal segment (near to duodenum) is not dilated.

***Experiment 2a: “Detection of extrahepatic biliary collaterals after tBDT at POD 21 and 28”*** ***(n* =* 10)***

Experiment 2a was dedicated to determine biliary collaterals after tBDT at two time points of interest: at POD 21 or 28 (each with *n* = 5 animals).

***Experiment 2b: “Detection of extrahepatic biliary collaterals after 70%PHx or sPVL in tBDT at*** ***POD 21” (n* =* 10)***

Experiment 2b was designed to detect biliary collaterals after 70%PHx or sPVL afterwards the induction of complete cholestasis (tBDT) (each with *n* = 5 animals). The extended liver resection (70%PHx) or the selective portal vein ligation (sPVL) was performed at POD 14.

***Experiment 2c: Detection of extrahepatic biliary collaterals after completed “two-stage hepatectomy” in*** ***tBDT at*** ***POD 28” (n* =* 5)***

Experiment 2c aimed to detect biliary collaterals after completed “two-stage hepatectomy” in tBDT (*n* = 5) at POD 28.

### Animals

All surgical procedures were performed in inbred male Lewis Rats (Charles River, Germany) aged 9–10 weeks (body weight 250–280 g). Rats were fed a standard laboratory diet with water and rat chow *ad libitum* until harvest. Rats were kept in groups of 2–3 animals under constant environmental conditions with a 12 h light–dark cycle in a conventional animal facility using environmentally enriched type IV cages. All procedures and housing of the animals were carried out according to the German Animal Welfare Legislation and approved by the local authorities (Landesamt für Verbraucherschutz, Reg.-Nr. 02-025/13).

#### Perioperative Preparation of the Animals

All animals were weighed and anesthetized with 3% isoflurane and 0.5 L/min oxygen in an induction chamber. During the operation the anesthesia was maintained with 2.5% isoflurane and 0.5 L/min oxygen using a inhalation mask for rats. The abdomen was shaved, and animals were placed in a supine position on a small animal operation table and fixed with tape. The abdominal skin was disinfected with iodine solution. A sterile operation field was created by placing sterile gauzes around the disinfected skin. A transverse incision was made in the upper third of the abdomen. Closure of the abdominal wound was always done by two-layer running suture.

### Laboratory Measurements

#### Liver Enzymes and Systemic Parameters

Blood samples were taken from the infrahepatic Vena cava and filled into special small blood tubes containing either sodium citrate (for coagulation tests) or serum separation gel (for clinical parameters). The blood tubes were stored at −20°C until measurement. After defreezing, the supernatants after centrifugation (2,000/min^−1^ for 3 min) were kept at crushed ice and used for measurement of the activities of alkaline phosphatase (AP; [U/L]), aspartate-aminotransferase (ASAT; [U/L]) and alanine-aminotransferase (ALAT; [U/L]), gamma-glutamyl-transferase [U/L], and content of albumin [g/dl], bilirubin [mg/dl], glucose [mmol/l], prothrombin time [%] in an automated chemical analyser (Bayer Advia 1650, Germany).

### Determination of the Weight and Volume of Liver Lobes

The liver lobes were weighed after explantation using an analytical balance (BLC-3000, Boeco Germany). The volume of the liver lobes was always determined with the method of displacement of tonnage (in sterilized distilled water), using calibrated graduated cylinder (Brand GmbH & Co.KG, Germany). Every sample was measured three times. The mean of the values was used for statistical evaluations.

Remnant liver body weight ratio was calculated by dividing the weight of the remnant liver [g] by the starting body weight [g] of the animal.

For better understanding, we included the volume data of an untreated animal (male Lewis rat, body weight 275 g) of our laboratory in the Supplementary Table 2 as time-point “0 = no OP” (whole liver volume: 8 ml, volume of “resected lobes”: 5 ml, volume of “FLR”: 3 ml).

### Histology and Immunohistochemistry

A sample from each liver lobe was taken according to a standardized protocol assuring evaluation of identical areas of the liver lobes in all animals. After staining, all slides were digitalized using a slide scanner (Nanozoomer, Hamamatsu Electronic Press Co., Ltd, Lwata, Japan).

We used Haematoxylin-eosin staining (HE) for histologic and morphological analysis of the liver tissue. We performed immunohistochemical staining to visualize the Bromodeoxyuridine (BrdU) incorporated in the newly synthesized nuclear DNA of proliferating hepatocytes and cholangiocytes (24).

Detailed descriptions of staining methods are listed in Supplementary Material.

#### Quantification of Proliferation

Proliferative activity of hepatocytes and cholangiocytes were determined using the HistoKat software developed at Fraunhofer Mevis (Dr. Homeyer, Fraunhofer MEVIS, Bremen, Germany). This software can be trained to either include or exclude elements in an image and is suitable for batch analysis of large numbers of images. The software was kindly provided by Fraunhofer-Institute (Fraunhofer MEVIS, Bremen, Germany) (25).

### *Ex vivo* Micro-Computed Tomography Imaging (μCT-Scan)

Digital images were taken from the explanted whole en-bloc samples using a digital single lens reflex camera (Canon EOS 450D + Canon EFS 18-55mm 1:3.5-5.6) and a digital stereo microscope (Leica M60 + IC80HD, Leica, Germany). Thereafter, the samples were fixed in formalin and afterwards scanned in a μCT enabling a digital 3D-reconstruction of the contrasted collaterals using the IMALYTICS Preclinical software (26, 27).

### Statistical Analysis

The data are expressed as mean ± standard deviation (SD) if not indicated otherwise. The data were analyzed using SPSS (IBM SPSS 22 for Windows). Type of distribution was determined using the Kolmogorow–Smirnow test (including the correction of significance according to Lilliefors). As the test revealed a non-normal distribution, the data were analyzed using non-parametric tests (Kruskal–Wallis Test, Mann–Whitney-*U*-Test). Differences were considered significant if *p*-value of less than 0.05 (2-tailed) were obtained (NS: not significant).

## Results

**Animals tolerated the complex surgical sequence of three operations well with a low mortality (2.9%, 68/70)**. Despite the challenging sequence of three consecutive hepatobiliary procedures within 21 days, the animals tolerated the complex sequence well with a survival rate of 97.14% (68/70) in both experiments. Two animals of experiment 1 died at POD 22 (1x Sham, 1x sBDT; 2/45) due to overdosed anesthesia. The remaining animals showed an uncomplicated postoperative course after each of the three surgical procedures. In the cholestatic groups, the body weight loss reached a maximum of 10% on the second (sBDT) or third (tBDT) postoperative day. Sham-operated animals showed a significantly stronger weight gain until sacrifice compared to cholestatic groups (see Supplementary Figure 2).

We found some soft adhesions in all animals irrespective of the extent of biliary occlusion, mostly at POD 21 and 28. These soft adhesions did not result in any hostile conditions preventing any of the surgical operations (e.g., sPVL, 70%PHx). The soft adhesions were always easy to dissect using micro-scissors. Mostly, the adhesions were located between liver lobes and the intestine at POD 21, whereas we found more interlobar adhesions at POD 28. In addition, we found no frequently swelling or infectious complications in the transverse laparotomy wounds in all rats. No additional wound closure or wound debridement was needed in any of the animals.

### Effect of Biliary Occlusion

Systemic levels of bilirubin (total) were significantly increased after tBDT compared to sBDT and Sham on POD 14. In contrast, partial biliary occlusion (sBDT) did not lead to increased systemic levels of bilirubin (total). We found significantly increased levels of ALAT and ASAT in tBDT in comparison to sBDT and Sham on POD 14 only. We observed no differences regarding hepatic synthetic function (e.g., albumin, prothrombin time) between the groups on all time points (see Supplementary Table 1).

**Total biliary occlusion (tBDT) led to a significant increase in whole liver volume (2.4-fold) due to massive biliary proliferates until POD 14**. In contrast, partial biliary occlusion (sBDT) caused an increase in volume only in the biliary obstructed liver lobes, resulting in a minor gain of total liver volume (1.7-fold). Sham showed a minor gain of whole liver volume (1.27-fold) (see Supplementary Table 2).

After tBDT, the periportal hepatocellular tissue was replaced by massive ductular proliferates occupying about 15% of the parenchymal tissue at POD 14. As expected, in sBDT we found comparable biliary proliferates only in the biliary occluded liver lobes (“future resected lobes”) on POD 14 (see Supplementary Tables 3A,B and Supplementary Figures 3A,B).

### Liver Regeneration and Atrophy

**Figure 2:**
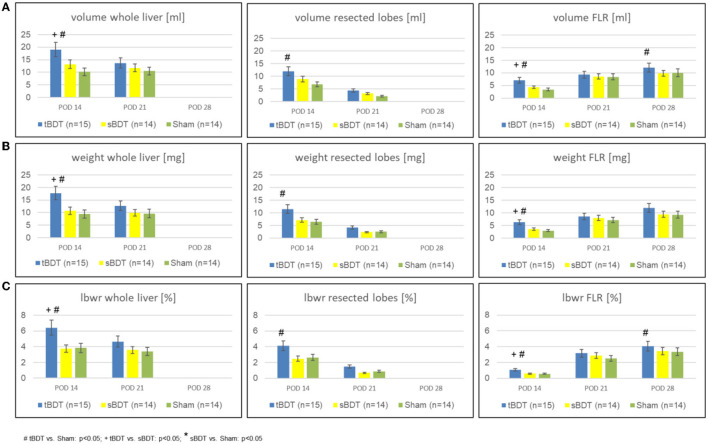
Variation of **(A)** volume, **(B)** weight, and **(C)** lbw-ratio of whole liver, resected lobes and FLR during the sequence “repeated regeneration stimuli” in different extents of biliary occlusion (tBDT 100%, sBDT 70%, Sham 0%) in rats (*n* = 45). We show the data for “whole liver”, the “future resected lobes” and for FLR of every group at the three time points of the sequence to illustrate the dynamic alterations of liver regeneration (# tBDT vs. Sham: *p* < 0.05; + tBDT vs. sBDT: *p* < 0.05; * sBDT vs. Sham: *p* < 0.05).

As expected, sPVL, caused a substantial volume gain (tBDT: 3-fold; sBDT: 2.8-fold; Sham 2.8-fold) of the future liver remnant (FLR) and a substantial volume loss (tBDT: 0.9-fold; sBDT: 0.6-fold; Sham: 0.4-fold) of the portally deprived “future resected lobes” compared to the preoperative liver volume. The subsequent 70%PHx promoted a further volume gain of the FLR in all groups (tBDT: 4-fold; sBDT: 3-fold; Sham 3-fold compared to original volume) until POD 28 (see Figures 2A–C and Supplementary Table 2).

Interestingly, in tBDT we always detected the strongest volume gain of FLR compared to sBDT and Sham on all time points, respectively.

### Hepatobiliary Remodeling

**Figure 3:**
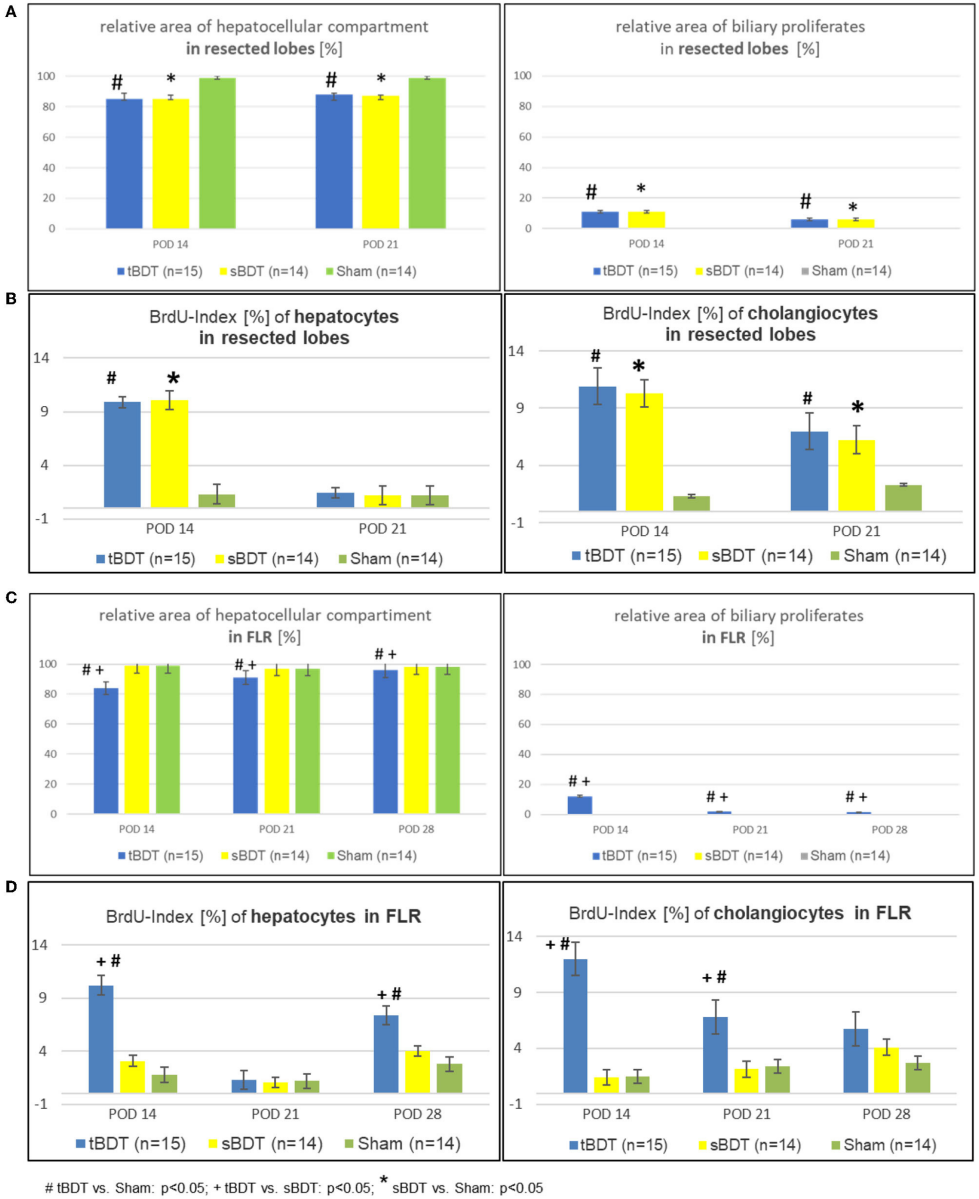
**(A–D)** Morphological alterations of the liver architecture focussing on the main cell compartments (e.g., hepatocytes and cholangiocytes) and their related proliferative activities during the sequence of “repeated regeneration stimuli” in **(A,B)** “resected lobes” and **(C,D)** the FLR during the sequence of “repeated regeneration stimuli” in different extents of biliary occlusion (tBDT 100%, sBDT 70%, Sham 0%) in rats (*n* = 45). The sequence consisted of two different regeneration stimuli: selective portal vein ligation at POD 14 (sPVL) followed by an extended liver resection at POD 21 (70%PHx) after induction of occlusive cholestasis of different extent (tBDT 100%, sBDT 70% or Sham with 0%; # tBDT vs. Sham: *p* < 0.05; + tBDT vs. sBDT: *p* < 0.05; * sBDT vs. Sham: *p* < 0.05).

**After tBDT**, we identified histologically three phases of hepatobiliary remodeling in the **FLR**. Following tBDT, biliary proliferates developed and replaced about 15% of the hepatocellular tissue. After sPVL, we found incomplete restoration of the hepatocellular tissue (FLR in tBDT ~9%) with a visible reduction of the biliary proliferates. The 70%PHx led to an almost complete recovery of the hepatocellular tissue in the FLR (tBDT ~4%) with a nearly normal liver architecture. In contrast, after sBDT and Sham we observed near normal liver morphology in the FLR at all time points (see Figures 3A–D, Supplementary Figures 3A,B, and Supplementary Tables 3A,B).

CT-scanning of the explanted livers and subsequent 3D reconstruction visualized the development of extrahepatic biliary collaterals. Collaterals were detected in 0/5 cases 1 week after sPVL (first regeneration stimulus), and in even more cases (3/5) 1 week after the 70%PHx (second regeneration stimulus). Histological workup identified the typical biliary cuboid epithelium as inner lining of the collaterals with the characteristic accompanying peribiliary glands (see Supplementary Tables 4, 5 and Supplementary Figures 4A–C).

**Histology and laboratory tests indicated a gradual biliary decompression** with decreasing levels of bilirubin to normal values and reduction of the biliary proliferates in FLR until 1 week after completed “two-stage hepatectomy” (see Supplementary Figure 5 and Supplementary Table 1). One week after 70%PHx we found a **substantial recovery of the hepatocellular mass** in a physiological hepatic architecture due to **formation of extrahepatic biliary collaterals** in the majority of the animals in tBDT (3/5, 60%). In contrast, after “tBDT only” or “tBDT plus one regeneration stimulus” (sPVL or 70%PHx) we found a biliary collateral only in the minority of the animals (15%, 3/20) (see Supplementary Table 4 and Supplementary Figures 4A–C). Furthermore, in tBDT the reduction of biliary proliferates was accompanied by a decreased proliferative activity of the cholangiocytes in FLR; and the recovery of the hepatocellular mass was accompanied by an increasing proliferative activity of the hepatocytes in FLR until POD 28 (see Supplementary Tables 3A,B and Supplementary Figures 3A,B).

## Discussion

Our study revealed two important findings: The proliferative pressure induced by the sequence of two regeneration stimuli promoted (1) restoration of biliary drainage via formation of extrahepatic biliary collaterals. The biliary decompression led finally to the (2) non-expected strong hepatocellular proliferation and visible volume gain of the FLR in animals subjected to tBDT.

The biliary decompression in our study was related to a non-intended formation of extrahepatic biliary collaterals in response to the proliferative forces of a sequence of two different regeneration stimuli. Biliary decompression after simple bile duct ligation (BDL) in rodents was described repeatedly. Several mechanisms were postulated. Some authors described a recanalization after ligating the common bile duct in 70–98% of the animals within 7–14 days or later (~20–28 days). Other authors reported about collateral biliary channels after BDL in rats (28–32). Since that time, the “ligation and transection technique” of the common bile duct (tBDT) has been accepted as standard technique for induction of a persistent biliary occlusion (8, 12, 14, 15, 33). In our study, biliary occlusion alone or with a single regeneration stimulus (sPVL or 70%PHx) resulted only in few animals in formation of biliary collaterals at late time points. In contrast, the sequence of two regeneration stimuli (sPVL+70%PHx) resulted in formation of biliary collaterals in the majority of the animals at early time points. Hence, the frequency and time point of formation of extrahepatic biliary collaterals seem to be associated with the number and intensity of additional regeneration stimuli. The histological workup of our samples revealed cholangiocytes as forming epithelial layer of the collaterals. In addition, the collaterals were accompanied by peribiliary glands, corresponding to the typical histological characteristics of bile ducts. The peribiliary glands were described as proliferative niche for cholangiocytes of the extrahepatic bile ducts (16–19, 34). Since we found an increased frequency of biliary collaterals after a sequence of two different regeneration stimuli, we propose that the collaterals were formed by cholangiocytes from peribiliary glands. We believe that the increased intraductal pressure alone or a single regenerative stimulus is not sufficient to promote the proliferation of cholangiocytes of the extrahepatic bile duct in rodents. Since biliary collaterals were not described in patients yet, our data are primarily important for a better understanding of biliary proliferation and remodeling in experimental models in rodents (1–7, 17–22, 35–38). At this point we cannot explain these differences between human and rat. In clinical routine, patients with a cholestatic altered liver are often not completely treated with a “two-staged hepatectomy” due to an insufficient volume gain of the FLR after sPVL or even the primarily marginal liver function prevents any intervention. Currently the number of reports increases about benefits of a preoperative biliary decompression (namely of the FLR) in cholestatic patients. Maybe these two differences in treatment of patients could serve as basic explanation for the non-detection of biliary collaterals in such highly selected patients (36–38).

The literature describes a substantial volume gain of the FLR as the prerequisite for timing the curative liver resection. Most authors postulate a volume gain of FLR after portal vein ligation on more than 30% of former whole liver volume. Less volume gain is considered to harbor an increased risk of liver failure due to an insufficient hepatic regenerative capacity (1–7). The experimental literature described maintenance and restoration of liver size (~volume) as the driving force and limitation of (acute) liver regeneration, especially after liver resection. Most of the authors equalized the restored liver mass with the recovery of the hepatocellular compartment (8, 12–16, 23, 39). As expected, our data of volume gain and histology of the FLR in sBDT and Sham were similar to the data found in the literature (1, 2, 6, 7, 16). In contrast, in tBDT the visible massive volume gain of the FLR was related primarily to biliary proliferates due to the biliary occlusion. While subsequent the stimuli, we found an impressive morphological alteration to a physiologic liver architecture with a persistent strong volume gain in FLR in tBDT. Strictly speaking, only the volume gain of FLR in tBDT following the stimuli can be considered as “macroscopic sign of liver regeneration” in tBDT facing the histologically proven recovery of the hepatocellular mass. Interestingly, the classical laboratory tests of hepatic synthetic function showed no evidence for a limited synthetic liver function, unless an elevated bilirubin due to the biliary obstruction in tBDT. Especially in experimental setting, the assessment of the functional capacity of the hepatocellular parenchyma of the FLR after volume gain due to regeneration stimuli is still problematic. We performed no excretory function tests since these tests are not standardized basing on their limited practicability and reliability of their results in rodents.

Hence, protection and preservation of the functional hepatocellular mass appears to evolve to a critical key aspect in multidisciplinary therapy concepts of liver tumors especially in occlusive cholestasis. However, the current literature provides no comparative histological analysis of liver tissue during a two-stage hepatectomy in cholestasis (41–48). We found few articles describing morphologically stronger distorted hepatocytes in FLR subsequent ALPPS (*A*ssociating *L*iver *P*artition with *P*ortal vein ligation for *S*taged hepatectomy) compared with two-stage hepatectomy in cholestatic or non-cholestatic patients (35, 40). The authors considered a preserved functional capacity of FLR in their patients as crucial and more important than the visible volume gain of the future liver remnant.

## Conclusion

The proliferative pressure of a sequence of two different regeneration stimuli (sPVL and liver resection) led to a non-expected biliary remodeling with biliary decompression and enabled the restoration of hepatocellular architecture in FLR in tBDT. Hence, this experimental model seems to be appropriate for further investigation of hepatobiliary remodeling and regeneration mechanisms in a cholestatic altered liver architecture.

## Data Availability Statement

The original contributions presented in the study are included in the article/Supplementary Material, further inquiries can be directed to the corresponding author/s.

## Ethics Statement

The animal study was reviewed and approved by German Animal Welfare Legislation (local authority: Landesamt für Verbraucherschutz, Thuringia, Tennstedter Straße 8/9, 99947 Bad Langensalza).

## Author Contributions

BR and UD designed the study. BR performed the study, made the 3D-reco of the μCT-Scans, analyzed the data, and wrote the manuscript. CS, FM, HS, UD, and US revised the manuscript. UD and US financed the study and publication. All authors contributed to the article and approved the submitted version.

## Conflict of Interest

The authors declare that the research was conducted in the absence of any commercial or financial relationships that could be construed as a potential conflict of interest.

## Publisher's Note

All claims expressed in this article are solely those of the authors and do not necessarily represent those of their affiliated organizations, or those of the publisher, the editors and the reviewers. Any product that may be evaluated in this article, or claim that may be made by its manufacturer, is not guaranteed or endorsed by the publisher.
