# Supplementary material for: The Interplay Between Biliary Occlusion and Liver Regeneration: Repeated Regeneration Stimuli Restore Biliary Drainage by Promoting Hepatobiliary Remodeling in a Rat Model
Source: Front Surg. 2022 Apr 25;9:799669. doi: 10.3389/fsurg.2022.799669 (PMC9081651; doi:10.3389/fsurg.2022.799669)
Supplement: Supplementary file 1 [file Data_Sheet_1.docx]

**Supplement**

**Material and Methods:**

*Preoperative preparation of the animals:*

All animals were weighed and anaesthetized with 3% isoflurane and 0.5 L/min oxygen in an induction chamber. The abdomen was shaved, and animals were placed in a supine position on a small animal operation table and fixed with tape. The anaesthesia was maintained as described above. The abdominal skin was disinfected with iodine solution. A sterile operation field was created by placing sterile gauzes around the disinfected skin. A transverse incision was made in the upper third of the abdomen. Closure of the abdominal wound was always done by two-layer running suture (Prolene 6-0, Ethicon).

*Postoperative care and analgesic treatment of the animals:*

Analgesic treatment was started immediately after the wound closure in all animals. Buprenorphine (0.05mg/kg BW, Temgesic®) was injected subcutaneously; twice per day for the first three postoperative days. During this time the animals were checked for their clinical condition also twice per day; afterwards the animals were routinely checked once per day. Clinical scoring was performed according to Hawkins [15]. For postoperative monitoring the rats were weighed daily.

**Surgical techniques**

All surgical interventions were performed at day-time under inhalation of 2-3% isoﬂurane mixed with pure oxygen at a flow rate of 0.5L/min (isoflurane vaporizer, Sigma, UK) in a dedicated S1 operation room. All instruments were thoroughly cleaned and tip-sterilized between the operations. At the end of the day the instruments were cleaned and sterilized in a commercial autoclave (Systec, Germany). All procedures were done using an operating microscope (Zeiss, magnification 10-25x, Germany) to ensure preservation of the branches of the hepatic artery and portal vein.

**Experimental groups**

*First operation: sBDT (selective ligation and transection of the left bile duct, draining 70% of liver) or tBDT (ligation and transection of the main bile duct, 100%) or Sham-manipulation at POD 0:*

For *sBDT* (~70% cholestasis) three silk ligatures were placed (silk 6-0) around the left bile duct draining the median and the left lateral lobe before transection of the left bile duct between the middle and distal ligature. The median lobe (ML) and the left lateral lobe (LLL) account for 70% of liver volume. The non-ligated, remaining liver lobes consisted of the right lobes (RL) and caudate lobes (CL) and represented the future liver remnant (FLR, ~30% of liver volume) [16, 46].

For *tBDT* (~100% cholestasis) three ligatures were placed around the main extrahepatic bile duct ca. 1cm above the pancreas. The ligated main extrahepatic bile duct was transected between the middle and most distal ligature.

In the sham operation, the main extrahepatic bile duct and the left bile duct were completely separated without placing ligatures or transecting the bile duct.

To facilitate the second operation (sPVL) the left branch of the portal vein (supplying ML and LLL) was identified and two ligatures were preplaced around the portal vein branch without tying. Instead, the ends of the ligatures were placed at the surface of LLL.

*Second operation at POD 14: sPVL (selective portal vein ligation)*

After relaparotomy, the surface of the left liver (ML, LLL) was exposed, the ends of the preplaced ligatures were identified and closed by a double surgical knot.

*Third operation at POD 21 (in experiment 1) and second operation at POD 14 (in experiment 2): 70%PHx (extended liver resection of 70% of liver, ML+LLL)*

After relaparotomy, the median liver lobe (consisting of right median lobe (RML) and left median lobe (LML)) and the left lateral liver lobe were resected after clamping. Subsequently 3-4 piercing sutures were placed in the base of the lobes in sufficient distance (3-4mm) from the cava to prevent outflow obstruction [46].

*Sacrifice at PODs 14, 21, 28 in Experiment 1:*

After relaparotomy, blood samples were taken from the infrahepatic vena cava. the animals were sacrificed by exsanguation under anaesthesia. The liver was explanted, weighed and the volume of the remnant liver and single liver lobes were determined. Samples of all liver lobes were collected for histological and immunohistochemical analysis according to a standardized protocol.

*Sacrifice at PODs 14, 21, 28 in Experiment 2 a-c:*

One hour prior to sacrifice the animals were injected with 50mg BrdU/kg body weight. After relaparotomy blood samples (EDTA, serum) were taken and animals were sacrificed by exsanguation under anaesthesia. The dilated proximal part of the ligated bile duct was exposed prior to placing a cannula (Vasofix® G 22 Braun, Germany). After draining the bile from the dilated duct, 3ml of Microfil© were slowly injected manually into the bile duct. The medium was allowed to polymerize for 24hrs at 4°C. Afterwards the liver was explanted en-bloc with the extrahepatic bile duct, duodenum and stomach and fixed in formalin and stored at 4°C until imaging took place.

***Haematoxylin-eosin staining (HE)***

The samples were fixed in 4.5% buffered formalin for 48h. Sections of 4μm thickness were cut after paraffin embedding. Slides were stained with Haematoxylin-Eosin (HE) for histo-pathological examination. After staining, all slides were digitalized using a slide scanner (Nanozoomer 2.0 HT scanner and the software NDP.scan 2.3; Hamamatsu City, Japan).

Number, size and relative area of necrotic areas or abscesses were evaluated with the measuring tool of NPG-Viewer (“NanoZoomer Digital Pathology”; Hamamatsu, Japan). Results for the number of necrotic areas were given as numerical value, for size in mm², and relative size in %. The relative area represents the area of necrosis in relation to area of the total section [%].

Number and relative area of periportal fields as well as the ductular reaction (bile ducts in portal area and extraportal convolutes of bile ducts) were determined and given as described for necrotic areas.

***Bromdesoxyuridin (BrdU Staining)***

The staining procedure based on a modified protocol of Sigma Inc. After deparaffinization and rehydration, tissue sections were treated with prewarmed 0.1% trypsin solution at 37°C for 20 minutes, followed by denaturation with 2 N HCl at 37°C for 30 minutes, and blocking with avidin solution for 10 minutes, biotin solution for 10 minutes, and 5% goat serum BSA-TBS at 37°C for 15 minutes. In the next step sections were incubated with 1:50 monoclonal anti-BrdU antibody (DAKO Inc.) at 37°C for 1 hour, followed by 1:300 biotinylated Fab-specific goat anti-mouse linked antibody (Sigma Inc.) for 30 minutes and AP-conjugated streptavidin (DAKO Inc.) for 30 minutes, prior to the application of Neofuchsin solution for 20 minutes. The sections were washed, counterstained with Hematoxylin, and coverslipped with Immu-Mount (Shandon Inc.).

**Results:**

**List of Figures in Manuscript:**

**Figure 1**: Design of Experiment 1 with the sequence of the “two-stage hepatectomy” in different extents of biliary occlusion (tBDT 100%, sBDT 70%, Sham 0%) in rats (n=45).

**Figure 2 A-C:** Variation of **(A)** volume, **(B)** weight and **(C)** lbw-ratio of whole liver, resected lobes and FLR during the sequence “repeated regeneration stimuli” in different extents of biliary occlusion (tBDT 100%, sBDT 70%, Sham 0%) in rats (n=45).

**Figure 3 A-D:** Morphological alterations of the liver architecture focussing on the main cell compartments (e.g., hepatocytes and cholangiocytes) and their proliferative activities in **(A+B)** “resected lobes” and **(C+D)** the FLR during the sequence of “repeated regeneration stimuli” in different extents of biliary occlusion (tBDT 100%, sBDT 70%, Sham 0%) in rats (n=45).

**List of Supplement Tables (each in an extra file):**

**Supplement Table 1:** Clinical Chemistry during the “two-stage hepatectomy” in different extents of biliary occlusion (BDT 100%, sBDT 70%, Sham 0%) in rats.

**Supplement Table 2:** Data of liver volume, weight and RLBW-ratio and relative volume gain of whole liver, “resected lobes” and FLR during the “two-stage hepatectomy” in different extents of biliary occlusion (BDT 100%, sBDT 70%, Sham 0%) in rats.

**Supplement Table 3 A:** Results of Morphometry and Immunohistochemistry (BrdU-Indices) of “**resected lobes**” during the “two-stage hepatectomy” in different extents of biliary occlusion (BDT 100%, sBDT 70%, Sham 0%) in rats.

**Supplement Table 3 B:** Results of Morphometry and Immunohistochemistry (BrdU-Indices) of “**FLR**” during the “two-stage hepatectomy” in different extents of biliary occlusion (BDT 100%, sBDT 70%, Sham 0%) in rats.

**Supplement Table 4:** Formation of biliary collaterals in relation to procedure and regeneration stimulus in experiments 2 a-c.

**Supplement Table 5:** Situs pictures and confirmation of biliary collaterals (blue arrows) by visualization on Macro-Pictures and 3D-Reco of scanned samples.

**List of Supplement Figures (each in an extra file):**

**Supplement Figure 1:** Design of experiment 2 a-c to detect biliary collaterals after tBDT only (2a), and after (2b) extended liver resection (70%PHx) or selective portal vein ligation (sPVL), and after (2c) “repeated regeneration stimuli” in style of a “two-stage hepatectomy” in tBDT. In all animals we injected coloured radiopaque Microfil© into the dilated proximal part of the ligated and transected bile duct at the time-point of sacrifice. The coloured and radiopaque Microfil© (MV-120) enabled macroscopic visualization and 3D-Reconstruction of the extrahepatic bile duct and biliary collaterals.

**Supplement Figure 2:** Postoperative body weight recovery. Similar body weight gain was observed in both cholestatic groups (sBDT with 70% and BDT 100% biliary occlusion). Sham operated animals had a significantly stronger weight gain compared to sBDT and BDT throughout the observation period.

**Supplement Figure 3A:** Comparison of Histology (HE) and Immunohistochemistry (BrdU) of **“resected lobes”** during “two-stage hepatectomy” in different extents of biliary occlusion (tBDT 100%, sBDT 70%, Sham 0%) in rats (n=45). Due to the biliary occlusion of the “resected lobes” in sBDT and tBDT we found a similar cholestatic liver parenchyma until POD 21 (see Supplement Tables 3A and 3B for detailed data). The proliferative activity of hepatocytes in either cholestatic group was similar increased at POD 14 followed by a decrease to physiological values at POD 21. Whereas the BrdU-levels of cholangiocytes showed significantly elevetad levels at both time points, compared to Sham respectively. Sham showed a normal hepatic architecture of “resected lobes” with physiological proliferative activities of hepatocytes and cholangiocytes.

**Supplement Figure 3B:** Comparison of Histology (HE) and Immunohistochemistry (BrdU) of **FLR** during “two-stage hepatectomy” in different extents of biliary occlusion (tBDT 100%, sBDT 70%, Sham 0%) in rats (n=45). Sham and sBDT showed a physiological hepatic architecture in FLR during the oberservation period. The most impressive morphological change was found in FLR in tBDT. Due to biliary occlusion we determined a massive ductular reaction with simultaneously reduction of the hepatocellular compartment by 15% at POD 14. Subsequent sPVL we detected a minor biliary decompression; but after 70%PHx we found a nearly physiological liver architecture with substantial recovery of the hepatocellular compartement in FLR until POD 28. The proliferative activity (BrdU) of hepatocytes in FLR in tBDT showed two peaks: at POD 14 and POD 28. The first peak reflected the repair mechanism of the hepatocellular damage due to the biliary occlusion. The second peak of BrdU-Levels revealed the proliferative burst of the recovering hepatocellular compartment subsequent the major liver resection. The decreasing BrdU-Levels of cholangiocytes in the cholestatic FLR (tBDT) following the regeneration stimuli, might be signalling the biliary decompression with declining proliferative stimulation of the cholangiocytes. In sBDT and Sham we found - as expected - physiologic BrdU-activities of hepatocytes and cholangiocytes in FLR.

**Supplement Figure 4A-C:** Histological workup of coloured biliary collaterals connecting the ligated and transected proximal part with the ligated distal part of the common bile duct after a sequence of different regeneration stimuli in rats with occlusive cholestasis (n=25). (**A):** One exemplary sample after completed “two-stage hepatectomy” in tBDT at POD 28. The red rectangle marks the area of the tissue sample for the **(B):** histological workup of the coloured collateral, the surrounding tissue, and the duodenum with particles of Microfil© (the black bar represents 2.5mm). **C:** Representative histological workup of the biliary collateral (bc) in relation to duration of tBDT and regeneration stimulus (10x and 40x magnification). All biliary collaterals showed typical characteristics of bile ducts with cholangiocytes as epithelial layer and peribiliary glands (black arrow). The biliary collateral was always surrounded by pancreatic tissue, fat tissue and connective tissue. We did not identify any lymphatic vessels or pancreatic duct as collaterals. The green histocolour assured identical alignment of the histological samples on the slides.

**Supplement Figure 5A-B:** Level of serum Bilirubin (total) during (A) the sequence of repeated regeneration stimuli in style of a “two-stage hepatectomy” **i**n different extents of biliary occlusion (tBDT 100%, sBDT 70%, Sham 0%) in rats (n=45) and **(B)** after BDT only in rats (n=5 per time points, respectively). **A:** We observed a gradually decrease in serum bilirubin during the sequence in the BDT animals with minor elevated levels at POD 28. The arrows in red signalling the time points of the first (sPVL) and the second regeneration stimulus (70%PHx). **B:** In contrast, rats subjected to tBDT only maintained high levels of serum bilirubin throughout the observation period of 28 days.
